# Chronic hepatitis infection is associated with extrahepatic cancer development: a nationwide population-based study in Taiwan

**DOI:** 10.1186/s12885-016-2918-5

**Published:** 2016-11-08

**Authors:** Abram Bunya Kamiza, Fu-Hsiung Su, Wen-Chang Wang, Fung-Chang Sung, Shih-Ni Chang, Chih-Ching Yeh

**Affiliations:** 1 School of Public Health, College of Public Health and Nutrition, Taipei Medical University, No. 250 Wu-Hsing Street, Taipei, 11031 Taiwan; 2 Department of Family Medicine, School of Medicine, College of Medicine, Taipei Medical University, No. 250 Wu-Hsing Street, Taipei, 11031 Taiwan; 3 Department of Family Medicine, Taipei Medical University Hospital, No. 252 Wu-Hsing Street, Taipei, 11031 Taiwan; 4 Master Program in Long-Term Care, College of Nursing, Taipei Medical University, No. 250 Wu-Hsing Street, Taipei, 11031 Taiwan; 5 School of Medicine, Flinders University, Bedford Park, Australia; 6 The Ph.D. Program for Translational Medicine, College of Medical Science and Technology, Taipei Medical University, No. 250 Wu-Hsing Street, Taipei, 11031 Taiwan; 7 Management Office for Health Data, China Medical University Hospital, No. 2 Yude Road, Taichung, 40402 Taiwan; 8 Graduate Institute of Clinical Medical Science, School of Medicine, College of Medicine, China Medical University, No. 91 Hsueh-Shih Road, Taichung, 40402 Taiwan; 9 Department of Public Health, China Medical University, No. 91 Hsueh-Shih Road, Taichung, 40402 Taiwan

**Keywords:** Hepatitis B virus, Hepatitis C virus, Cancer risk, Taiwan

## Abstract

**Background:**

Hepatitis B virus (HBV) and hepatitis C virus (HCV) are the major causes of chronic hepatitis infection (CHI). This longitudinal cohort study investigated the association of CHI with hepatic and extrahepatic cancer development in Taiwan.

**Methods:**

Patients with HBV infection and HCV infection were identified from the Taiwan National Health Insurance Research Database. A Cox proportional hazard model was used to calculate hazard ratios (HRs) and 95 % confidence intervals (CIs) for determining the association between CHI and cancer development.

**Results:**

The patients with HBV infection exhibited an increased risk of colorectal cancer (HR: 1.36, 95 % CI: 1.09–1.70), liver cancer (HR: 21.47, 95 % CI: 18.0–25.6), gallbladder and extrahepatic bile duct cancer (HR: 2.05, 95 % CI: 1.07–3.91), pancreatic cancer (HR: 2.61, 95 % CI: 1.47–4.61), kidney cancer (HR: 1.72, 95 % CI: 1.10–2.68), ovarian cancer (HR: 2.31, 95 % CI: 1.21–4.39), and non-Hodgkin’s lymphoma (HR: 2.10, 95 % CI: 1.25–3.52). The patients with HCV infection exhibited an increased risk of liver cancer (HR: 25.10, 95 % CI: 20.9–30.2), gallbladder and extrahepatic bile duct cancer (HR: 2.60, 95 % CI: 1.42–4.73), ovarian cancer (HR: 5.15, 95 % CI: 1.98–13.4), and non-Hodgkin’s lymphoma (HR: 2.30, 95 % CI: 1.34–3.96).

**Conclusion:**

The present population-based study revealed that in addition to its association with primary liver cancer, CHI is associated with an increased risk of extrahepatic cancer.

## Background

Hepatitis B virus (HBV) and hepatitis C virus (HCV) are the major causes of chronic hepatitis infection (CHI). Approximately 2 billion people worldwide have been infected with HBV, and 360 million people are currently chronic carriers [1]. HCV has been estimated to infect approximately 185 million people worldwide, with the highest prevalence in Central and East Asian, North African, and Middle Eastern regions [2], and more than 75 % of chronic HBV carriers reside in Asian countries, including Taiwan [3]. The prevalence of hepatitis B surface antigen (HBsAg) carriers in Asia is estimated to be 8–12 % [4]. Patients with CHI are at an increased risk of liver fibrosis, liver cirrhosis, and hepatocellular carcinoma [5, 6].

Epidemiological studies have reported an association between CHI and primary liver cancer development [6–9]. Furthermore, some studies have revealed an association between CHI and the development of extrahepatic cancers such as pancreatic cancer [10], gallbladder and extrahepatic bile duct cancer [11], intrahepatic cholangiocarcinoma, and non-Hodgkin’s lymphoma [12–15]. A study in Sweden reported an association between chronic HBV infection and upper aerodigestive tract, lung, kidney, skin, and thyroid gland cancers; lymphoma; and leukemia [16]. However, a case–control study in Shanghai, China, demonstrated that patients with HBV had no risk of cancers of the gallbladder, ampulla of Vater, and bile duct [17]. Overall, data on the association between CHI and extrahepatic cancer development in countries with endemic HBV and HCV infection are lacking. Previous studies have been conducted in countries with low prevalence and endemicity; hence, drawing a statistically supported conclusion from their results is difficult [15, 17, 18]. Moreover, these studies have focused on the association of HBV or HCV with primary liver cancer; comprehensive data on extrahepatic cancers among patients with CHI are lacking.

This longitudinal cohort study comprehensively investigated the association of CHI with extrahepatic cancer development in Taiwan, using a nationwide population-based data set. HBV infection is endemic and HCV infection is highly prevalent in Taiwan [19]. In addition, cancer is highly prevalent in Taiwan, making the country an excellent setting for studying the association of CHI with cancer.

## Methods

### Data sources

In this study, the Longitudinal Health Insurance Database 2000 (LHID2000) of the National Health Insurance (NHI) program, which was launched in March 1995 to provide affordable healthcare services to all residents of Taiwan, was used. The program covered 93 % of the population in 1997, and the coverage rate increased to approximately 99.9 % by the end of 2014. The National Health Insurance Research Database (NHIRD) is a nationwide database extracted from the claims data of the NHI program for research purposes. This database contains information on inpatient and outpatient medical claims, including prescription and diagnosis records.

The LHID2000, which is a data set of the NHIRD, contains the claims data of one million beneficiaries randomly selected from all of the residents enrolled in the NHI program in 2000. No significant differences have been observed in age, sex, or healthcare costs between the entire population of this data set and all beneficiaries of the NHI program. Approval to use all the claims data and updated registries in the LHID2000 from 2000 to 2011 was received, and the International Classification of Diseases, Ninth Revision, Clinical Modification (ICD-9-CM) was used to identify disease diagnoses in the NHIRD. All data were anonymized upon inclusion in the NHIRD. Notably, this study was exempted from full review by the Institutional Review Board at China Medical University and the Hospital Research Ethics Committee (IRB permit number: CMU-REC-101-012).

### Study sample

**Fig. 1:**
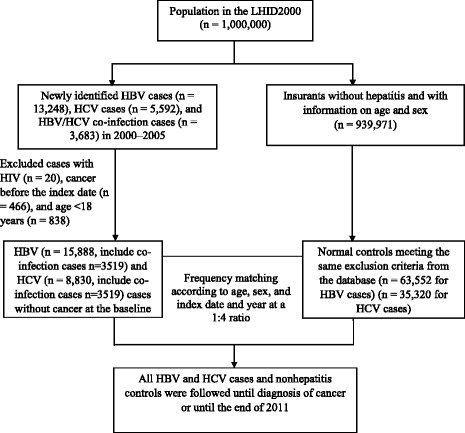
Flowchart of patient recruitment

In this study, the association of CHI with hepatic and extrahepatic cancer development among an adult population (≥18 years old) was investigated. The etiologies of other types of chronic hepatitis, such as autoimmune, chemical, and alcohol-related hepatitis, and nonalcoholic fatty liver disease, were excluded. Additionally, the presence of HBsAg was used as the major serum marker for cases of HBV infection recorded in the database. Patients with a history of human immunodeficiency virus (HIV) were excluded to minimize the inclusion of patients with HBV/HIV coinfection with occult HBV infection (i.e., HBsAg-negative patients with persistent HBV infection) [20]. Therefore, patients with a history of HIV (ICD-9-CM codes 042, 043, 044, V08, and 795.8) and chronic hepatitis (ICD-9-CM codes 571.4, 571.8, 571.9, and 573.3) without mention of HBV (ICD-9-CM codes 070.2, 070.3, and V02.61) or HCV (ICD-9-CM codes 070.41, 070.44, 070.51, 070.54, and V02.62) infection were excluded. The index date for patients with chronic HBV or HCV infection was the first date on which chronic HBV or HCV infection was detected. Patients with a diagnosis of cancer (ICD-9-CM codes 140–208) before the index date were also excluded. After applying the exclusion criteria, 15,888 patients with HBV infection (including 3,519 coinfected with HCV) and 8,830 with HCV infection (including 3,519 coinfected with HBV), who were identified during 2000–2005, were enrolled in this study as the CHI cohort and followed up until cancer diagnosis or the end of 2011, whichever occurred first. In total, 939,971 insurants without hepatitis and with information on age and sex were identified; after excluding those aged < 18 years who had had HIV or cancer before the index date, 63,552 and 35,320 control participants were identified and included in the non-HBV cohort and non-HCV cohort, respectively. The nonhepatitis cohorts were frequency matched to the CHI cohort at a ratio of 4:1 by age, sex, and index date and year (Fig. 1).

Patients newly diagnosed with head and neck cancer (ICD-9-CM codes 140 and 149), esophageal cancer (ICD-9-CM code 150), stomach cancer (ICD-9-CM code 151), colorectal cancer (ICD-9-CM codes 153 and 154), liver cancer (ICD-9-CM code 155), gallbladder and extrahepatic bile duct cancer (ICD-9-CM code 156), pancreatic cancer (ICD-9-CM code 157), lung cancer (ICD-9-CM code 162), melanoma (ICD-9-CM code 172), skin cancer (ICD-9-CM code 173), breast cancer (ICD-9-CM codes 174 and 175), uterine and corpus cancer (ICD-9-CM codes 179 and 182), cervical cancer (ICD-9-CM code 180), ovarian cancer (ICD-9-CM code 183), prostate cancer (ICD-9-CM code 185), bladder cancer (ICD-9-CM code 188), kidney cancer (ICD-9-CM code 189), brain cancer (ICD-9-CM code 191), thyroid cancer (ICD-9-CM code 193), non-Hodgkin’s lymphoma (ICD-9-CM code 202), myeloma (ICD-9-CM code 203), and leukemia (ICD-9-CM codes 204 and 208) during 2000–2011 were identified from the Registry of Catastrophic Illness Patients. Insurance coverage for catastrophic illnesses is an extension of the NHI program that protects people with serious disease against a devastating financial burden and subsequent impoverishment.

### Statistical analyses

Pearson’s chi-square test was used to compare the distributions of sociodemographic factors and various comorbidities, such as diabetes mellitus, hypertension, and hyperlipidemia, between the CHI cohort and the nonhepatitis cohorts, and the Student *t*-test was used to compare the number of outpatient visits between the CHI cohort and the nonhepatitis cohorts. Urbanization was categorized into four levels, with level 1 referring to the most urbanized communities and level 4 to the least urbanized communities. The geographical regions where the patients resided were divided into Northern Taiwan, Central Taiwan, Southern Taiwan, Eastern Taiwan, and the outlying islands. Additionally, the patients’ monthly incomes were categorized into four groups: NT$0, NT$1–NT$15,840, NT$15,841–NT$25,000, and > NT$25,000.

The cancer incidence rates were evaluated from the initial follow-up to the end of 2011. The follow-up period (years) was defined as the duration from chronic viral hepatitis identification to cancer diagnoses or censoring for death, emigration, or withdrawal from the NHI program, whichever occurred first. Poisson regression was used to calculate the incidence rate ratios with 95 % confidence intervals (CIs) for comparison of our HBV or HCV cohorts with the adult population in the LHID2000. Finally, a Cox proportional hazard model was used to calculate hazard ratios (HRs) and 95 % CIs for determining the association between CHI and cancer development. HRs were adjusted for sex, age, geographical region, occupation, level of urbanization, monthly income, the presence of comorbidities, and number of outpatient visits. A p value < 0.05 was considered statistically significant. All statistical analyses were performed using SAS (Version 9.4 for Windows; SAS Institute, Inc., Cary, NC, USA).

## Results

In our study, the patients with HBV infection were more likely to be laborers, reside in Central and Southern Taiwan, and have a higher monthly income, compared with the patients without HBV infection (Table 1). Moreover, these patients were more likely to have diabetes mellitus, hypertension, and hyperlipidemia. Similarly, the patients with HCV infection were more likely to be laborers, reside in less urbanized areas in Southern Taiwan, and have comorbidities, compared with the patients without HCV infection.

**Table 1:** Baseline characteristics and comorbid conditions in hepatitis cohorts identified in 2000–2005

	HBV							HCV						
Variable	No *n* (%)		Yes *n* (%)		*X* ^2^	df	*p* value	No *n* (%)		Yes *n* (%)		*X* ^2^	df	*P* value
Sex					0.00	1	1.000					0.00	1	1.000
Women	26832	(42.2)	6708	(42.2)				16864	(47.7)	4216	(47.7)			
Men	36720	(57.8)	9180	(57.8)				18456	(52.3)	4614	(52.3)			
Age, years					0.00	3	1.000					0.00	3	1.000
< 50	45564	(71.7)	11391	(71.7)				17116	(48.5)	4279	(48.5)			
50–59	9308	(14.6)	2327	(14.6)				7284	(20.6)	1821	(20.6)			
60–69	5896	(9.3)	1474	(9.3)				6784	(19.2)	1696	(19.2)			
≥ 70	2784	(4.4)	696	(4.4)				4136	(11.7)	1034	(11.7)			
Geographical region					212.15	3	<0.0001					967.58	3	<0.0001
Northern	30226	(47.6)	6594	(41.5)				16176	(45.8)	2666	(30.2)			
Central	12404	(19.5)	3401	(21.4)				6962	(19.7)	1882	(21.3)			
Southern	16079	(25.3)	4698	(29.6)				9110	(25.8)	3597	(40.7)			
Eastern and islands	4843	(7.6)	1195	(7.5)				3072	(8.7)	685	(7.8)			
Occupation					118.28	4	<0.0001					277.96	4	<0.0001
Public	5956	(9.4)	1829	(11.5)				3519	(10.0)	758	(8.6)			
Labor	19351	(30.4)	5159	(32.5)				12796	(36.2)	4010	(45.4)			
Business	30002	(47.2)	7070	(44.5)				14066	(39.8)	2879	(32.6)			
Low income	256	(0.4)	65	(0.4)				183	(0.5)	68	(0.8)			
Retired	7987	(12.6)	1765	(11.1)				4756	(13.5)	1115	(12.6)			
Urbanization level					94.02	3	<0.0001					428.06	3	<0.0001
1 (highest)	19662	(30.9)	4431	(27.9)				10499	(29.7)	1873	(21.2)			
2	18754	(29.5)	4713	(29.7)				10162	(28.8)	2531	(28.7)			
3	11896	(18.7)	2952	(18.6)				6314	(17.9)	1513	(17.1)			
4 (lowest)	13232	(20.8)	3791	(23.9)				8340	(23.6)	2912	(33.0)			
Monthly income, NT$					79.93	3	<0.0001					127.57	3	<0.0001
0	13698	(21.6)	3282	(20.7)				8233	(23.3)	1869	(21.2)			
1–15,840	7712	(12.1)	1633	(10.3)				4180	(11.8)	990	(11.2)			
15,841–25,000	27135	(42.7)	6795	(42.8)				15717	(44.5)	4495	(50.9)			
> 25,000	15007	(23.6)	4178	(26.3)				7190	(20.4)	1476	(16.7)			
Diabetes mellitus					232.42	1	<0.0001					280.01	1	<0.0001
No	59599	(93.8)	14355	(90.4)				31531	(89.3)	7311	(82.8)			
Yes	3953	(6.2)	1533	(9.6)				3789	(10.7)	1519	(17.2)			
Hypertension					88.26	1	<0.0001					122.42	1	<0.0001
No	54129	(85.2)	13054	(82.2)				26215	(74.2)	6038	(68.4)			
Yes	9423	(14.8)	2834	(17.8)				9105	(25.8)	2792	(31.6)			
Hyperlipidemia					347.37	1	<0.0001					121.67	1	<0.0001
No	57936	(91.2)	13702	(86.2)				30422	(86.1)	7194	(81.5)			
Yes	5616	(8.8)	2186	(13.8)				4898	(13.9)	1636	(18.5)			
Outpatient visits, mean (SD)	12	(13)	16	(15)			<0.0001^a^	14	(15)	21	(18)			<0.0001^a^

*X* ^2^ Chi-square test, *df* degree of freedom

^a^t-test

Table 2 presents the incidence densities of cancers among the patients with CHI. The Poisson regression model revealed that, compared with the adult population in the LHID2000, the patients with HBV or HCV infection exhibited an increased risk of liver cancer (HR: 12.89, 95 % CI: 11.9–13.9 or HR: 16.26, 95 % CI: 15.1–17.5, respectively). In addition, HBV infection was associated with an increased risk of developing thyroid gland cancer, stomach cancer, colorectal cancer, gallbladder and extrahepatic bile duct cancer, pancreatic cancer, lung cancer, kidney cancer, bladder cancer, uterine and corpus cancer, ovarian cancer, prostate cancer, breast cancer, skin cancer, non-Hodgkin’s lymphoma, and leukemia. Similarly, HCV infection was associated with an increased risk of developing head and neck cancer, stomach cancer, colon and rectum cancer, gallbladder and bile duct cancer, pancreatic cancer, lung cancer, kidney cancer, bladder cancer, uterine cancer, prostate cancer, skin cancer, and non-Hodgkin’s lymphoma.

**Table 2:** Incidence densities of cancers in patients diagnosed with chronic HBV and HCV infection

	All LHID2000			HBV				HCV		
Cancer type	Events	Rate^a^	Events	Rate^a^	IRR^b^	(95 % CI)	Events	Rate^a^	IRR^b^	(95 % CI)
Overall	40213	36.59	1330	94.01	2.95	(2.80–3.12)*	1232	163.00	3.33	(3.15–3.53)
Brain cancer	509	0.46	7	0.49	1.13	(0.53–2.38)	7	0.93	1.61	(0.76–3.39)
Head and neck cancer	4079	3.71	66	4.67	1.23	(0.97–1.57)	48	6.35	1.43	(1.08–1.90)*
Thyroid gland cancer	1138	1.04	24	1.70	1.78	(1.19–2.67)*	13	1.72	1.53	(0.89–2.65)
Esophageal cancer	850	0.77	15	1.06	1.45	(0.87–2.42)	11	1.46	1.48	(0.82–2.68)
Stomach cancer	2247	2.04	32	2.26	1.42	(1.00–2.01)*	37	4.90	1.78	(1.28–2.46)*
Colorectal cancer	5890	5.36	104	7.35	1.69	(1.39–2.05)*	83	10.98	1.51	(1.21–1.87)*
Liver cancer	5293	4.82	770	54.43	12.89	(11.9–13.9)*	782	103.46	16.26	(15.1–17.5)*
Gallbladder and extrahepatic bile duct cancer	501	0.46	14	0.99	2.80	(1.64–4.76)*	18	2.38	3.81	(2.38–6.10)*
Pancreatic cancer	684	0.62	19	1.34	2.73	(1.73–4.31)*	17	2.25	2.66	(1.65–4.31)*
Lung cancer	4656	4.24	74	5.23	1.57	(1.25–1.97)*	69	9.13	1.61	1.27–2.04)*
Kidney cancer	1065	0.97	28	1.98	2.47	(1.70–3.60)*	17	2.25	1.71	(1.06–2.76)*
Bladder cancer	1324	1.20	24	1.70	1.83	(1.22–2.74)*	24	3.18	1.98	(1.33–2.97)*
Uterine and corpus cancer^c^	600	1.10	14	2.29	2.12	(1.25–3.61)*	11	2.98	2.17	(1.19–3.93)*
Cervical cancer^c^	1877	3.43	10	1.64	0.51	(0.27–0.95)	11	2.98	0.62	(0.34–1.12)
Ovarian cancer^c^	592	1.08	15	2.45	2.29	(1.37–3.82)*	8	2.16	1.67	(0.83–3.35)
Prostate cancer^d^	1820	3.30	30	3.73	1.83	(1.28–2.63)*	24	6.21	1.55	(1.04–2.32)*
Breast cancer^c^	4695	8.58	74	12.10	1.43	(1.14–1.80)*	46	12.45	1.17	(0.87–1.56)
Melanoma	136	0.12	0	--	--	--	2	0.26	1.58	(0.39–6.37)
Skin cancer	619	0.56	15	1.06	2.44	(1.46–4.08)*	11	1.46	1.90	(1.04–3.44)*
Non-Hodgkin’s lymphoma	738	0.67	22	1.56	2.58	(1.69–3.94)*	20	2.65	3.01	(1.93–4.69)*
Myeloma	218	0.20	2	0.14	0.87	(0.22–3.49)	3	0.40	1.47	(0.47–4.60)
Leukemia	682	0.62	15	1.06	1.82	(1.09–3.04)*	7	0.93	1.19	(0.56–2.50)

*IRR* incidence rate ratio

^a^Per 10,000 person-years

^b^Adjusted for sex and age

^c^Women only

^d^Men only

*p< 0.05

Compared with their corresponding nonhepatitis cohorts, the overall adjusted HRs for the risk of various cancers were 2.67 (95 % CI: 2.49–2.86) and 2.83 (95 % CI: 2.63–3.05) for the HBV and HCV cohorts, respectively (Table 3). Specifically, the patients with HBV infection exhibited an increased risk of colorectal cancer (HR: 1.36, 95 % CI: 1.09–1.70), liver cancer (HR: 21.47, 95 % CI: 18.0–25.6), gallbladder and extrahepatic bile cancer (HR: 2.05, 95 % CI: 1.07–3.91), pancreatic cancer (HR: 2.61, 95 % CI: 1.47–4.61), kidney cancer (HR: 1.72, 95 % CI: 1.10–2.68), ovarian cancer (HR: 2.31, 95 % CI: 1.21–4.39), and non-Hodgkin’s lymphoma (HR: 2.10, 95 % CI: 1.25–3.52). Further analysis revealed that HCV was also a significant risk factor for liver cancer (HR: 25.10, 95 % CI: 20.9–30.2), gallbladder and extrahepatic bile duct cancer (HR: 2.60, 95 % CI: 1.42–4.73), ovarian cancer (HR: 5.15, 95 % CI: 1.98–13.4), and non-Hodgkin’s lymphoma (HR: 2.30, 95 % CI: 1.34–3.96).

**Table 3:** Hazard ratios for developing cancer in patients with CHI

	HBV				HCV			
	No	Yes			No	Yes		
Cancer type	Cases	Cases	HR	(95 % CI)^a^	Cases	Cases	HR	(95 % CI)^a^
Overall	2,139	1,330	2.67	(2.49–2.86)^‡^	1,893	1,232	2.83	(2.63–3.05)^‡^
Brain cancer	30	7	1.04	(0.45–2.39)	18	7	1.67	(0.68–4.15)
Head and neck cancer	288	66	0.96	(0.73–1.26)	171	48	1.13	(0.81–1.57)
Thyroid gland cancer	61	24	1.50	(0.93–2.42)	44	13	1.18	(0.62–2.24)
Esophageal cancer	54	15	1.42	(0.79–2.54)	46	11	1.24	(0.63–2.43)
Stomach cancer	132	32	1.11	(0.75–1.65)	122	37	1.41	(0.96–2.06)
Colorectal cancer	341	104	1.36	(1.09–1.70)^†^	327	83	1.07	(0.84–1.38)
Liver cancer	155	770	1.47	(18.0–25.6)^‡^	137	782	25.10	(20.9–30.2)^‡^
Gallbladder and extrahepatic bile duct cancer	30	14	2.05	(1.07–3.91)*	32	18	2.60	(1.42–4.73)^†^
Pancreatic cancer	35	19	2.61	(1.47–4.61)^†^	55	17	1.51	(0.86–2.65)
Lung cancer	292	74	1.11	(0.86–1.44)	318	69	0.93	(0.71–1.21)
Kidney cancer	69	28	1.72	(1.10–2.68)*	68	17	0.99	(0.57–1.71)
Bladder cancer	80	24	1.24	(0.78–1.97)	87	24	1.20	(0.75–1.90)
Uterine and corpus cancer^b^	43	14	1.28	(0.69–2.37)	26	11	1.82	(0.87–3.78)
Cervical cancer^b^	62	10	0.74	(0.38–1.46)	62	11	0.81	(0.42–1.56)
Ovarian cancer^b^	27	15	2.31	(1.21–4.39)*	10	8	5.15	(1.98–13.4)^†^
Prostate cancer^c^	134	30	0.95	(0.64–1.42)	136	24	0.81	(0.52–1.27)
Breast cancer^b^	256	74	1.25	(0.96–1.62)	197	46	1.08	(0.78–1.50)
Melanoma	14	0	--	--	12	2	0.78	(0.17–3.60)
Skin cancer	37	15	1.46	(0.79–2.69)	37	11	1.09	(0.54–2.17)
Non-Hodgkin’s lymphoma	45	22	2.10	(1.25–3.52)^†^	45	20	2.30	(1.34–3.96)^†^
Myeloma	12	2	0.93	(0.20–4.25)	13	3	1.15	(0.32–4.20)
Leukemia	43	15	1.44	(0.79–2.61)	25	7	1.21	(0.51–2.87)

*p < 0.05, ^†^ *p* < 0.001, ^‡^ *p* < 0.0001

^a^Adjusted for sex, age, geographical region, occupation, level of urbanization, monthly income, the presence of comorbidities, and number of outpatient visits

^b^Women only

^c^Men only

Furthermore, we analyzed our data after excluding those with HBV/HCV coinfection. The patients with only HBV infection exhibited an increased risk of colorectal cancer (HR: 1.51, 95 % CI: 1.15–1.98), liver cancer (HR: 18.9, 95 % CI: 15.2–23.6), kidney cancer (HR: 1.81, 95 % CI: 1.10–3.01), and non-Hodgkin’s lymphoma (HR: 2.22, 95 % CI: 1.18–4.18), whereas the patients with HCV exhibited an increased risk of liver cancer (HR: 23.28, 95 % CI: 18.4–29.5), gallbladder and extrahepatic bile duct cancer (HR: 2.53, 95 % CI: 1.17–5.48), and non-Hodgkin’s lymphoma (HR: 2.66, 95 % CI: 1.34–5.27) (Table 4).

**Table 4:** Hazard ratios for developing cancer in patients with CHI after excluding HBV/HCV coinfected patients

	HBV				HCV			
	No	Yes			No	Yes		
Cancer type	Cases	Cases	HR	(95 % CI)^a^	Cases	Cases	HR	(95 % CI)^a^
Overall	1,517	829	2.34	(2.15–2.55)^‡^	1,174	731	2.71	(2.47–2.99)^‡^
Brain cancer	21	4	0.85	(0.29–2.51)	13	4	1.51	(0.48–4.77)
Head and neck cancer	233	47	0.84	(0.61–1.16)	107	29	1.06	(0.70–1.62)
Thyroid gland cancer	53	20	1.41	(0.84–2.38)	23	9	1.62	(0.73–3.61)
Esophageal cancer	42	13	1.47	(0.78–2.76)	22	9	2.08	(0.93–4.65)
Stomach cancer	78	18	1.01	(0.60–1.69)	76	23	1.38	(0.85–2.24)
Colorectal cancer	212	72	1.51	(1.15–1.98)^†^	204	51	1.09	(0.79–1.49)
Liver cancer	102	439	18.9	(15.2–23.6)^‡^	86	451	23.28	(18.4–29.5)^‡^
Gallbladder and extrahepatic bile duct cancer	20	7	1.56	(0.65–3.73)	19	11	2.53	(1.17–5.48)*
Pancreatic cancer	33	13	1.69	(0.88–3.23)	32	11	1.68	(0.82–3.41)
Lung cancer	198	48	1.07	(0.78–1.47)	212	43	0.87	(0.62–1.21)
Kidney cancer	52	22	1.81	(1.10–3.01)*	47	11	0.98	(0.49–1.93)
Bladder cancer	67	13	0.87	(0.48–1.58)	65	13	0.91	(0.50–1.68)
Uterine and corpus cancer^b^	33	11	1.26	(0.63–2.50)	25	8	1.61	(0.71–3.67)
Cervical cancer^b^	42	10	1.10	(0.55–2.21)	32	11	1.52	(0.75–3.08)
Ovarian cancer^b^	23	8	1.35	(0.60–3.07)	10	1	0.58	(0.07–4.62)
Prostate cancer^c^	96	22	0.93	(0.58–1.49)	82	16	0.84	(0.48–1.46)
Breast cancer^b^	192	53	1.15	(0.84–1.56)	92	25	1.24	(0.78–1.95)
Melanoma	11	0	--	--	7	2	1.48	(0.29–7.56)
Skin cancer	27	13	1.90	(0.97–3.73)	24	9	1.35	(0.61–2.99)
Non-Hodgkin’s lymphoma	29	15	2.22	(1.18–4.18)*	27	13	2.66	(1.34–5.27)^†^
Myeloma	9	2	1.04	(0.22–4.90)	5	3	3.25	(0.73–14.5)
Leukemia	35	10	1.24	(0.61–2.52)	22	2	0.35	(0.08–1.53)

*p < 0.05, ^†^ *p* < 0.001, ^‡^ *p* < 0.0001

^a^Adjusted for sex, age, geographical region, occupation, level of urbanization, monthly income, the presence of comorbidities, and number of outpatient visits

^b^Women only

^c^Men only

## Discussion

The present population-based study revealed that CHI is associated with an increased risk of extrahepatic cancer in Taiwan. Approximately 15.4 % of the global cancer burden can be attributed to five infectious agents, namely Epstein–Barr virus, human papillomavirus, HBV, HCV, and *Helicobacter pylori* [21]. HBV, an enveloped DNA virus from the hepadnavirus family, has a high affinity for hepatocytes. In Asia, where HBV infection is highly endemic, vertical transmission is the main route of HBV exposure. By contrast, HCV is an RNA virus from the flavivirus family that is commonly transmitted horizontally through contaminated blood, blood products, and intravenous drug use.

The association between CHI and primary liver cancer has been extensively documented [7, 22–24], and in this study, the patients with CHI exhibited an increased risk of liver cancer. However, the mechanism by which hepatitis viruses cause liver cancer remains unclear. It has been suggested that hepatitis viruses cause genomic instability through integration into human chromosomes, which causes the chromosomal rearrangement of cellular genes and increases the likelihood of hepatocarcinogenesis [25]. Furthermore, patients with CHI are at an increased risk of non-Hodgkin’s lymphoma [12–15]. In this study, the patients with CHI exhibited a higher risk of non-Hodgkin’s lymphoma than the nonhepatitis cohorts. This finding supports and extends previous reports of a significant association between CHI and non-Hodgkin’s lymphoma. Moreover, the patients with CHI exhibited an increased risk of gallbladder and extrahepatic bile duct cancer, which confirms that patients with CHI are at an increased risk of cancer [11].

In addition to the well-established association between CHI and primary liver cancer, our results indicated that HBV infection is associated with an increased risk of pancreatic cancer. However, the association was nonsignificant after excluding the patients with HBV/HCV coinfection. This observation is attributed to the small sample size for pancreatic cancer; hence, statistical power was decreased. The pancreas serves as a potential reservoir of hepatitis viruses because of its close proximity to the liver, and the blood vessels and ducts it shares with the liver [26]. Thus, the increased risk of pancreatic cancer among the patients with HBV can be attributed to these two factors. Moreover, previous meta-analyses have reported an increased risk of pancreatic cancer among patients with HBV infection [27, 28], which corroborate our findings.

In the present study, other cancers such as kidney cancer, colorectal cancer, and ovarian cancer were also associated with CHI. Sundquist et al. determined that there was an increased incidence of kidney cancer among patients with HBV, substantiating our results [16]. Nevertheless, the increased risk of kidney cancer observed here is a novel finding that requires further investigation. Only a few studies have investigated the association between CHI and colorectal cancer, and they have reported inconsistent findings [18, 29]. Rustagi et al. demonstrated that HCV is an independent risk factor for colorectal adenoma, and reported that HCV is associated with a 2.04-fold higher risk of colorectal cancer [29]. By contrast, in this study, we only observed this association among the patients with HBV. In addition, previous studies have suggested that X protein from HBV can bind and interfere with the components of the DNA repair machinery and p53 tumor suppressor in response to DNA damage, thereby increasing the risk of colorectal cancer [18, 30]. In addition, the HBV X protein has been reported to be highly expressed in the ovarian cancer cells of Chinese women, implying that it may be involved in the carcinogenesis of ovarian cancer [31]. However, the association between CHI and the aforementioned cancers was nonsignificant after excluding the patients with HBV/HCV coinfection, which reduced the statistical power of the study.

By contrast, Mahale et al. and Lee et al. have argued that there is an increased risk of head and neck, prostate, and esophageal cancers among patients with HCV [32, 33]. However, a nonsignificant association was observed between CHI and these cancers in this study, which is consistent with the findings of similar research in the United States [34, 35]. This discrepancy can be attributed to the control group selected by Mahale et al., which included patients diagnosed with lung, esophageal, and urinary bladder cancers [32]. Moreover, the novel findings reported by Lee et al. can be attributed to the study’s small sample size for esophageal and prostate cancers [33].

A major strength of the present study is that it analyzed a large cohort of patients identified from the NHIRD, covering nearly the entire population of Taiwan. Only patients diagnosed with HIV infection, alcohol-related hepatitis, and autoimmune hepatitis were excluded, to prevent the confounding effects of these diseases from skewing our results. However, this study has some limitations. First, the NHIRD does not contain detailed information about patients’ smoking habits, alcohol consumption, family cancer history, body mass index, nutritional status, environmental exposure to chemicals, or history of substance use; therefore, we could not rule out the potential confounding effects of these factors. Another limitation is the small sample size for specific rare cancers, which reduced the statistical power of this study.

## Conclusions

In addition to the well-established association between CHI and primary liver cancer, the present population-based cohort study revealed that CHI is associated with an increased risk of extrahepatic cancers of the colon and rectum, gallbladder and extrahepatic bile ducts, pancreas, kidneys, and ovaries, as well as non-Hodgkin’s lymphoma.

## Abbreviations

CHI: Chronic hepatitis infection
CI: Confidence interval
HBsAg: Hepatitis B surface antigen
HBV: Hepatitis B virus
HCV: Hepatitis C virus
HIV: Human immunodeficiency virus
HR: Hazard ratio
ICD-9-CM: International classification of diseases, ninth revision, clinical modification
LHID2000: Longitudinal health insurance database 2000
NHI: National health insurance
NHIRD: National Health insurance research database
